# Supplementary figures and images for: Casein kinase 1 gamma regulates oxidative stress response via interacting with the NADPH dual oxidase complex
Source: PLoS Genet. 2023 Apr 26;19(4):e1010740. doi: 10.1371/journal.pgen.1010740 (PMC10166522; doi:10.1371/journal.pgen.1010740)

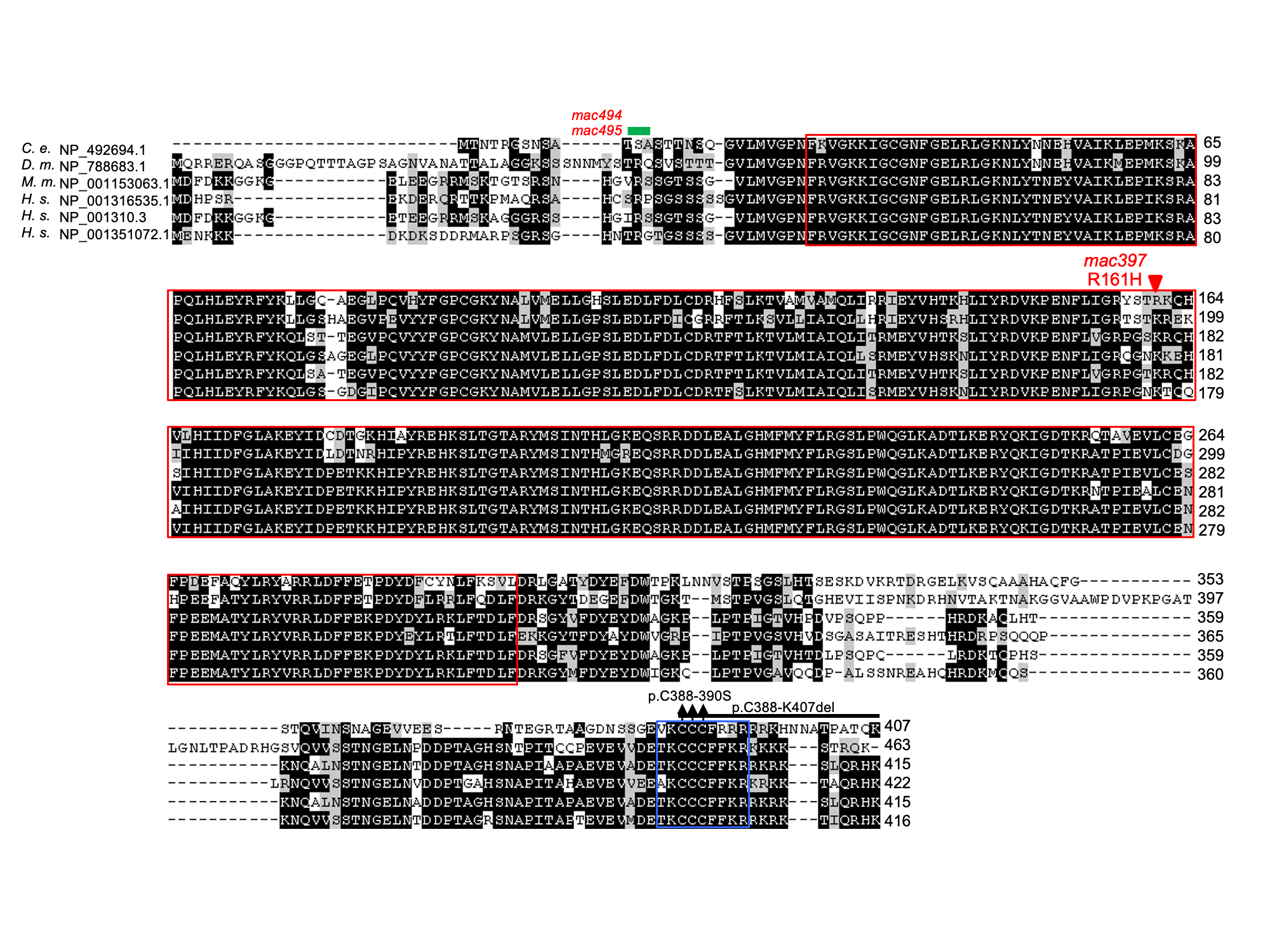

Supplement: S1 Fig — C.e.: C. elegans CSNK-1; D.m.: Drosophila Gilgamesh; M.m.: mouse CSNK1G2; H.s.: human CSNK1G1, CSNK1G2, CSNK1G3. Green bar: frameshift regions caused by mac494 and mac495 mutations. Red box: kinase domain. Red arrowhead: mac397 mutation. Blue box: conserved palmitoylation signal. (TIFF) [file pgen.1010740.s001.tiff]

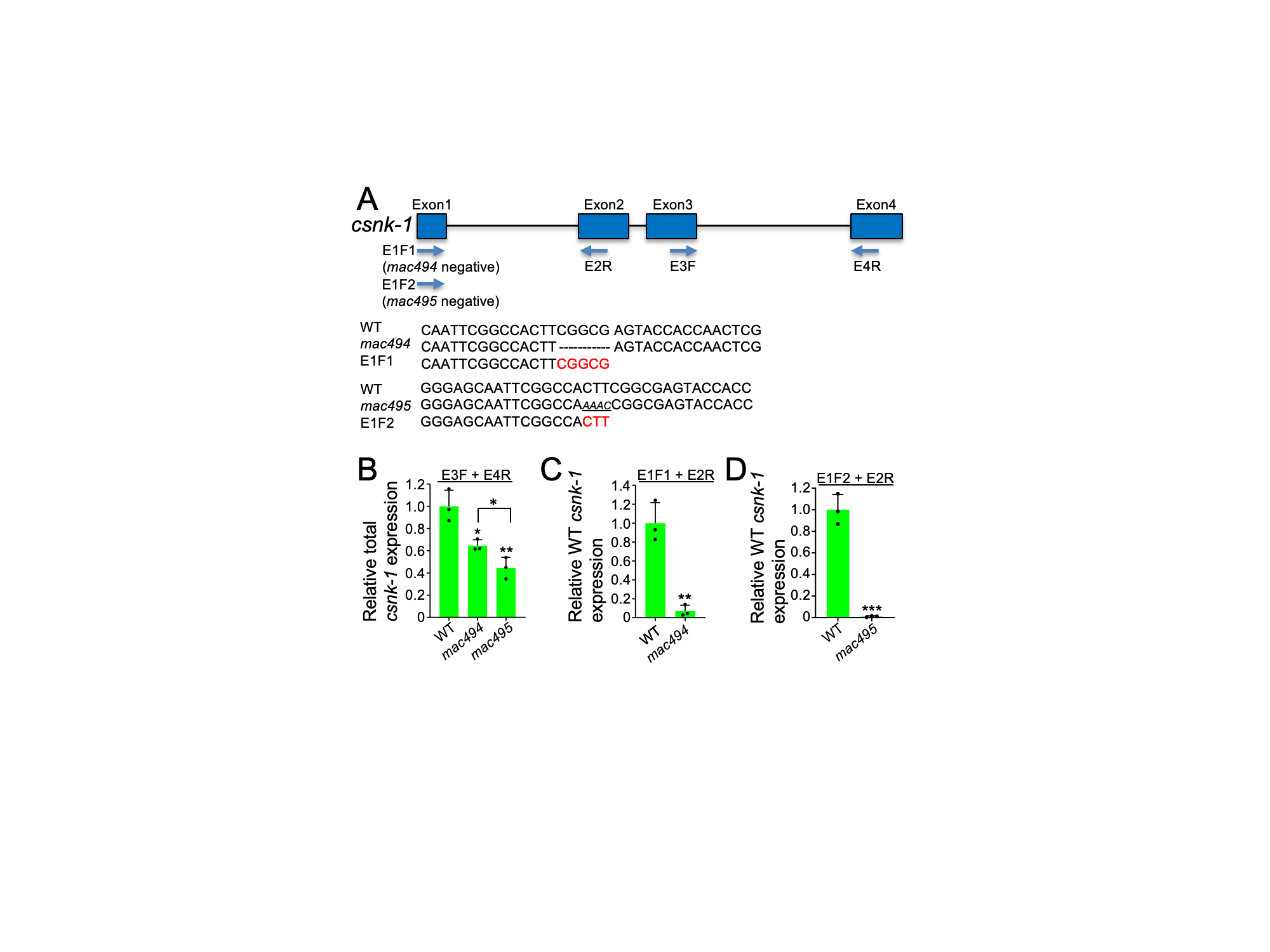

Supplement: S2 Fig — (A) Positions and sequences of PCR primers for detecting all or wildtype-only csnk-1 transcripts. Partial wildtype and mutant csnk-1 sequences are aligned to show the specificity of the primers for wildtype-only transcripts. (B) Relative total csnk-1 transcript levels. (C, D) Relative wildtype-only csnk-1 transcript levels in wildtype, csnk-1(mac494lf) or csnk-1(mac495lf) animals. tba-1 was the loading control. Statistics: two-tailed unpaired Student’s t-test. *: p < 0.05; **: p < 0.01; ***: p < 0.001. (TIFF) [file pgen.1010740.s002.tiff]

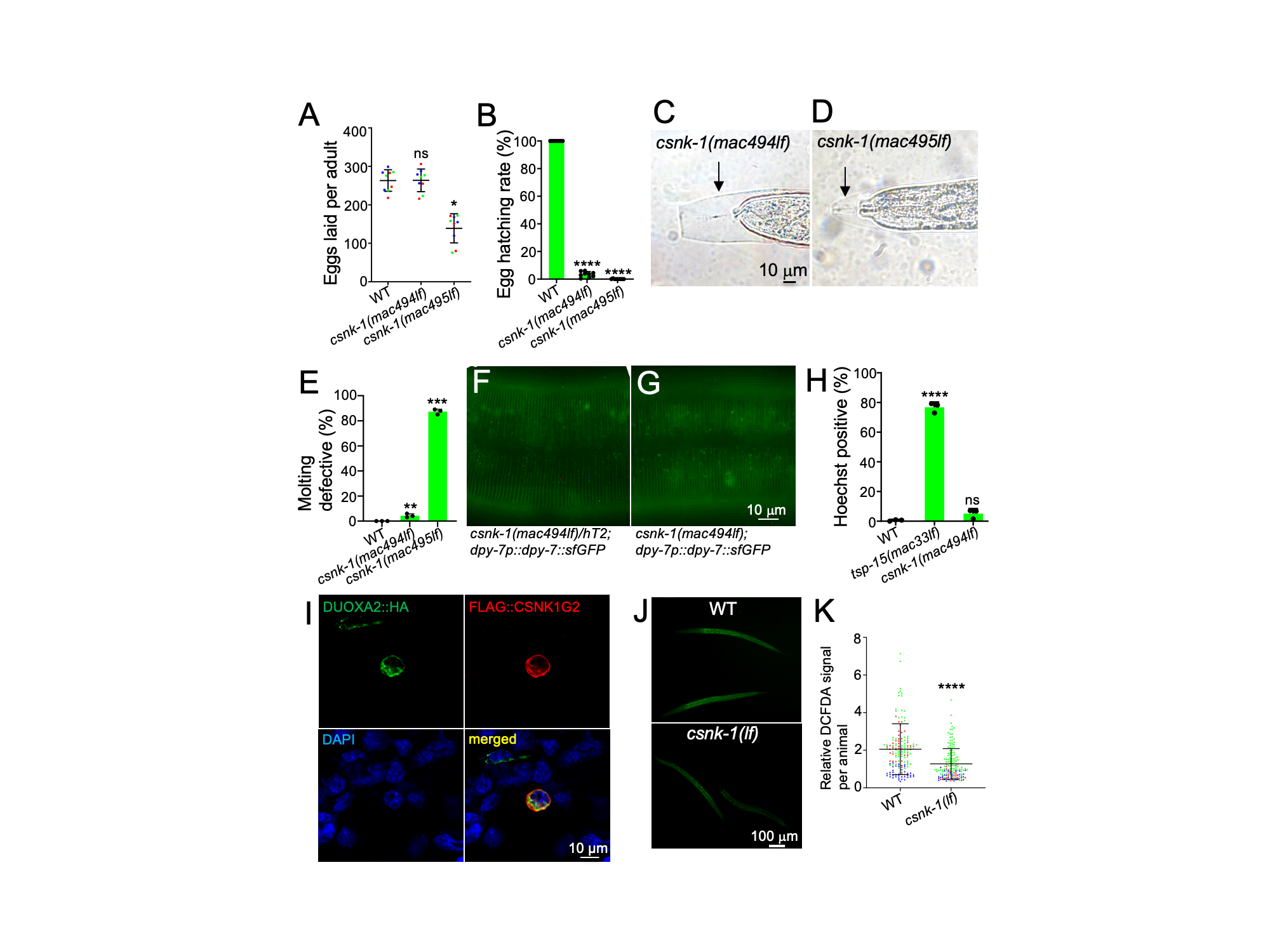

Supplement: S3 Fig — (A) Number of all eggs laid per adult. Results were based three biological replicates, with three animals per replicate. Colors represent different replicates. Statistics: two-tailed unpaired Student’s t-test. *: p < 0.05. ns: not significant. (B) Hatching rate of eggs laid by csnk-1(lf) homozygous mutants. All eggs laid by a single adult on regular NGM agar plates were examined. Results were based on three biological replicates, with three animals per replicate. Statistics: two-tailed unpaired Student’s t-test. ****: p < 0.0001. (C, D) Representative molting defects of csnk-1(lf) mutants. Arrows indicate attached cuticles. (E) Quantification of young adults (24 hrs after mid-L4 larval stage) with molting defects. Results were based on three biological replicates, with 100 animals analyzed in each replicate. Statistics: two-tailed unpaired Student’s t-test. **: p < 0.01; ***: p < 0.001. (F, G) Typical cuticle stripe patterns of csnk-1(lf)/+ and csnk-1(lf) mutants labeled by a DPY-7::sfGFP reporter. (H) Percentage of young adults positively stained by the nuclear dye Hoechst 33258. Results were based on three biological replicates, with 59–141 animals in each replicate. Statistics: two-tailed unpaired Student’s t-test. ****: p < 0.0001. ns: not significant. (I) Colocalization of overexpressed DUOXA2::HA and FLAG::CSNK1G2 in a HeLa cell. (J) Representative fluorescent pictures of wildtype and csnk-1(lf) L4 animals stained with DCFDA. Pictures were taken with the same exposure time of 600 ms and fluorescent intensity of each animal was measured using ImageJ. (K) Quantification of DCFDA fluorescent signals of individual L4 animals. Results were based on three biological replicates, with 21–100 animals in each replicate. Statistics: two-tailed unpaired Student’s t-test. ****: p < 0.0001. (TIFF) [file pgen.1010740.s003.tiff]

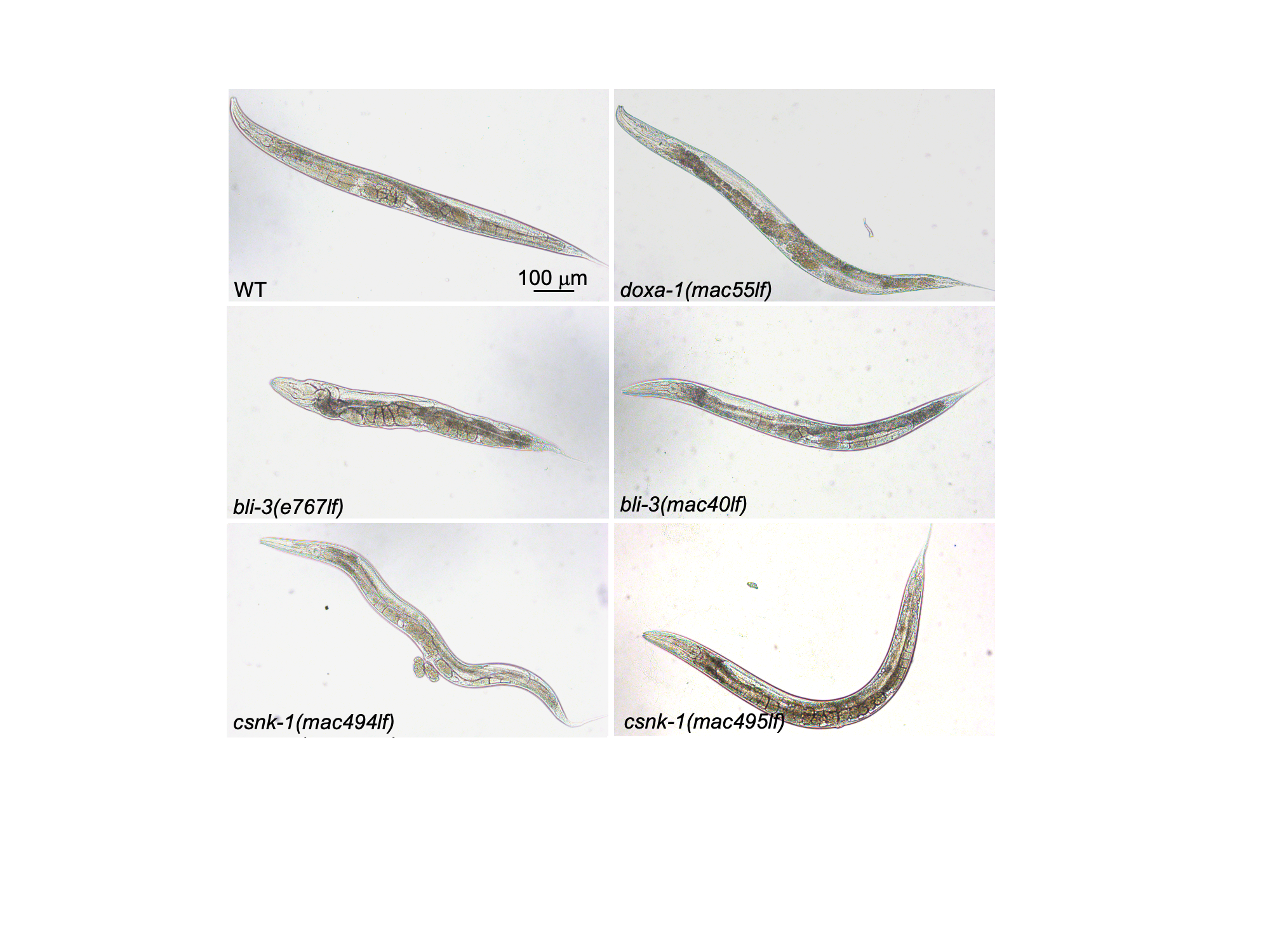

Supplement: S4 Fig — All images are of the same scale. (TIFF) [file pgen.1010740.s004.tiff]

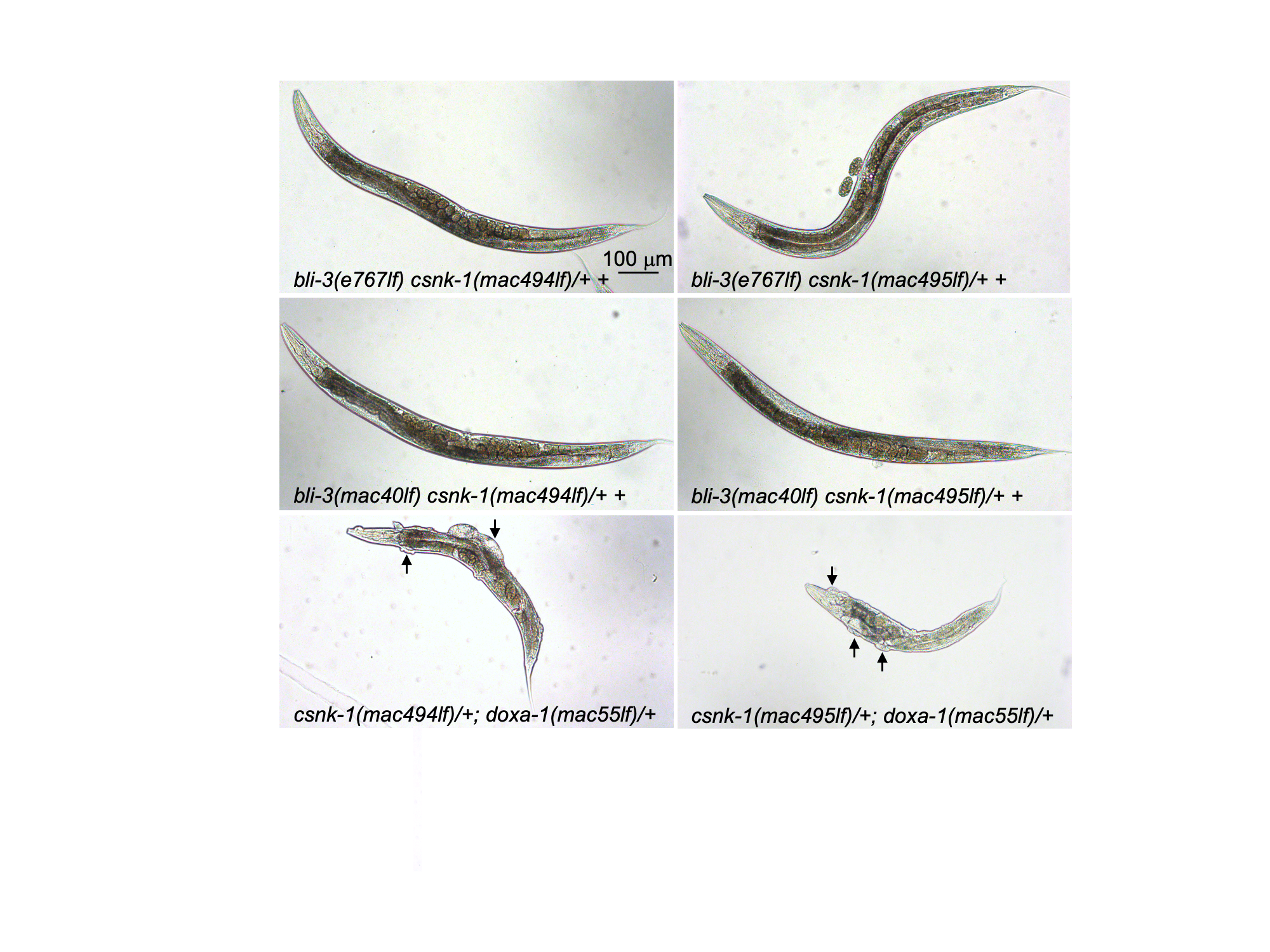

Supplement: S5 Fig — bli-3(lf) csnk-1(lf)/hT2 (treated as bli-3(lf) csnk-1(lf)/+ +) mutants had wildtype-like morphology, while csnk-1(lf)/hT2; doxa-1(lf)/hT2 (treated as csnk-1(lf)/+; doxa-1(lf)/+) mutants exhibited obviously blistered and dumpy phenotype. Arrows point to blisters. All images are of the same scale. (TIFF) [file pgen.1010740.s005.tiff]

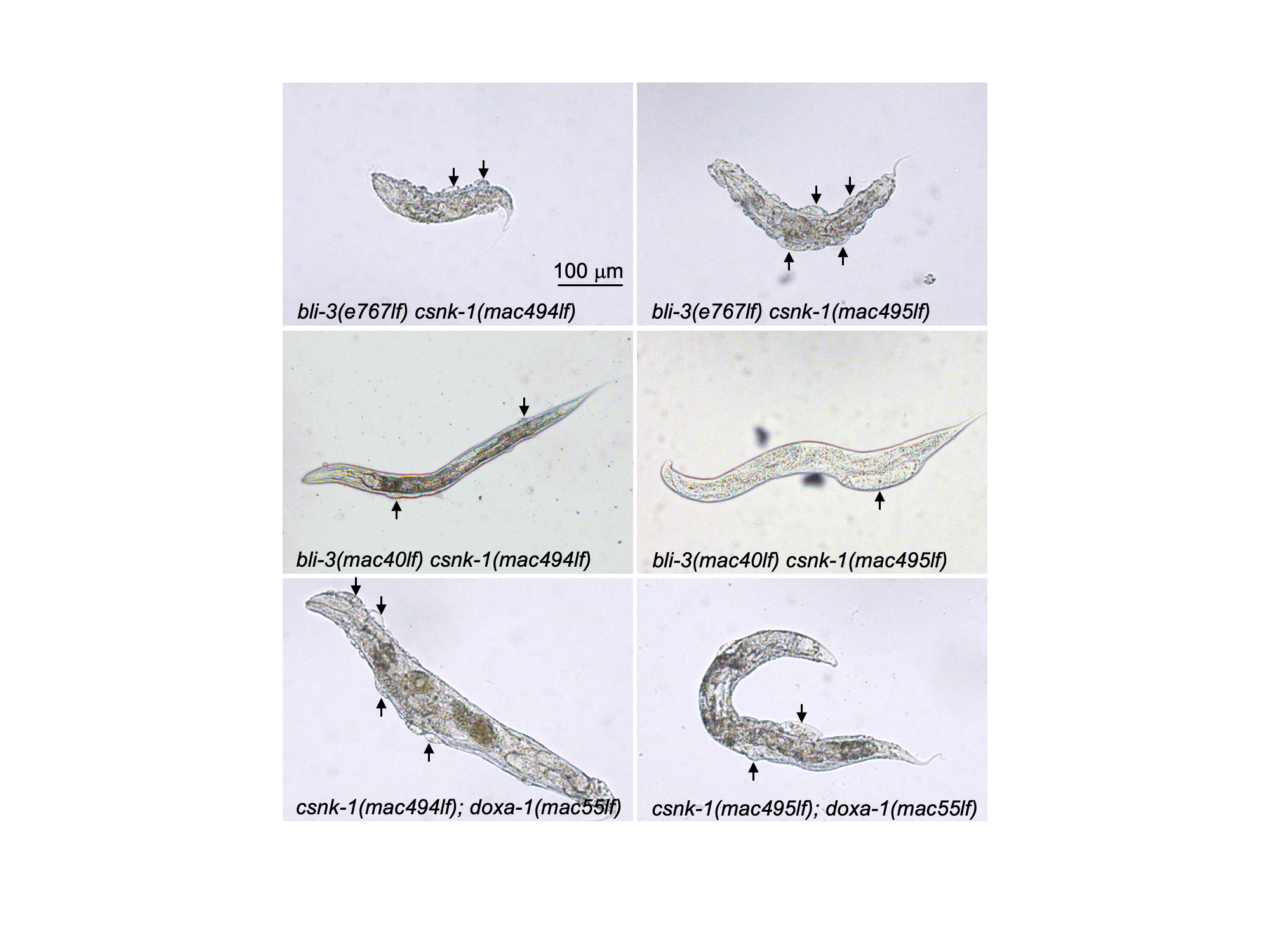

Supplement: S6 Fig — bli-3(lf) csnk-1(lf) double homozygous mutants were derived from bli-3(lf) csnk-1(lf)/hT2 heterozygous mutants. csnk-1(lf); doxa-1(lf) double homozygous mutants were derived from csnk-1(lf)/hT2; doxa-1(lf)/hT2 heterozygous mutants. Arrows point to typical blisters. All images are of the same scale. (TIFF) [file pgen.1010740.s006.tiff]

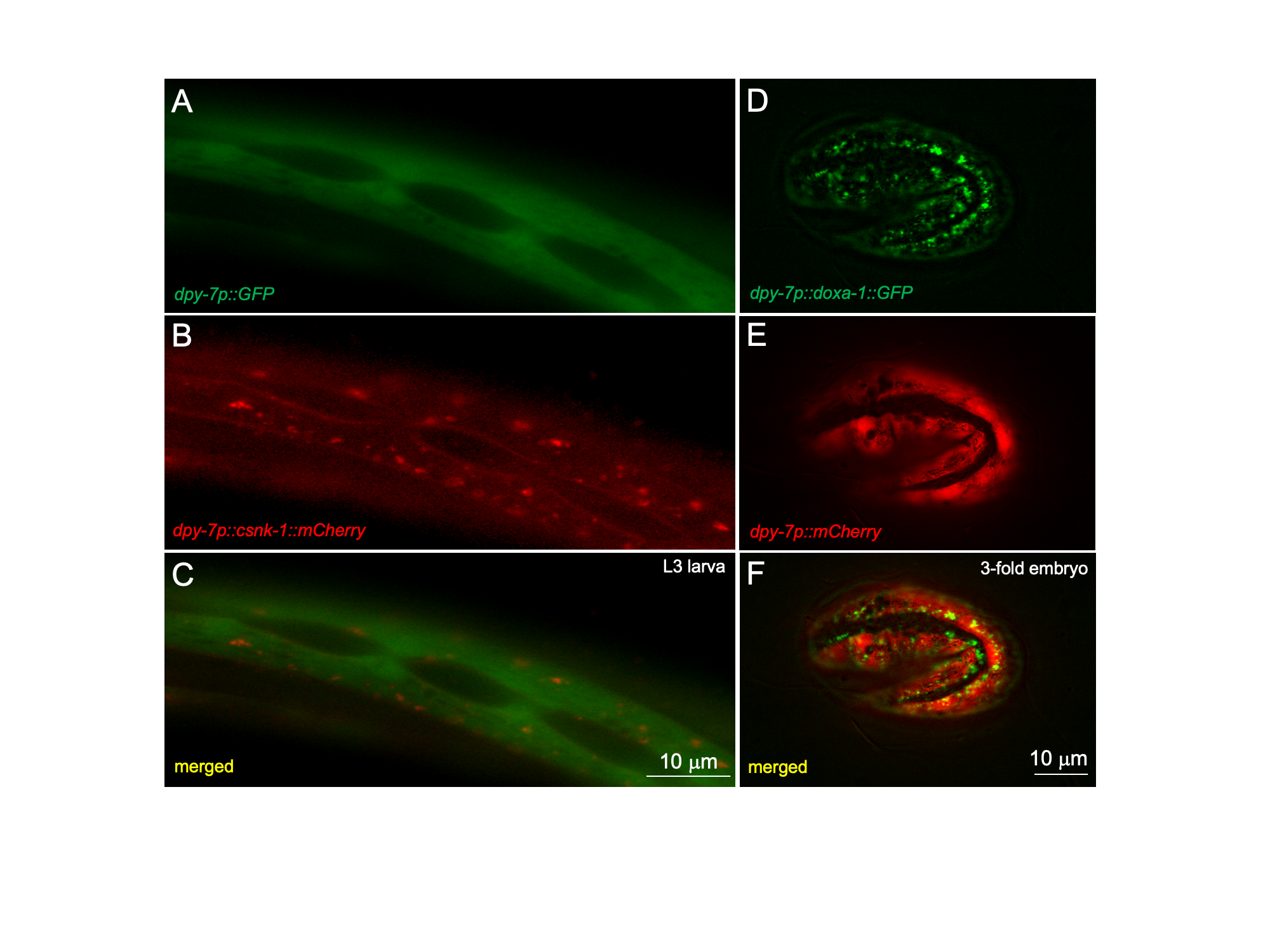

Supplement: S7 Fig — (A, B, C) A transgenic L3 larvae co-expressing GFP and CSNK-1::mCherry in epithelial cells. (D, E, F) A transgenic 3-fold embryo co-expressing DOXA-1::GFP and mCherry in epithelial cells. For unclear reason, DOXA-1::GFP was strongly expressed in embryos but was not visible at larval stages in these transgenic lines. We therefore observed whether DOXA-1::GFP colocalizes with mCherry in embryos. (TIFF) [file pgen.1010740.s007.tiff]

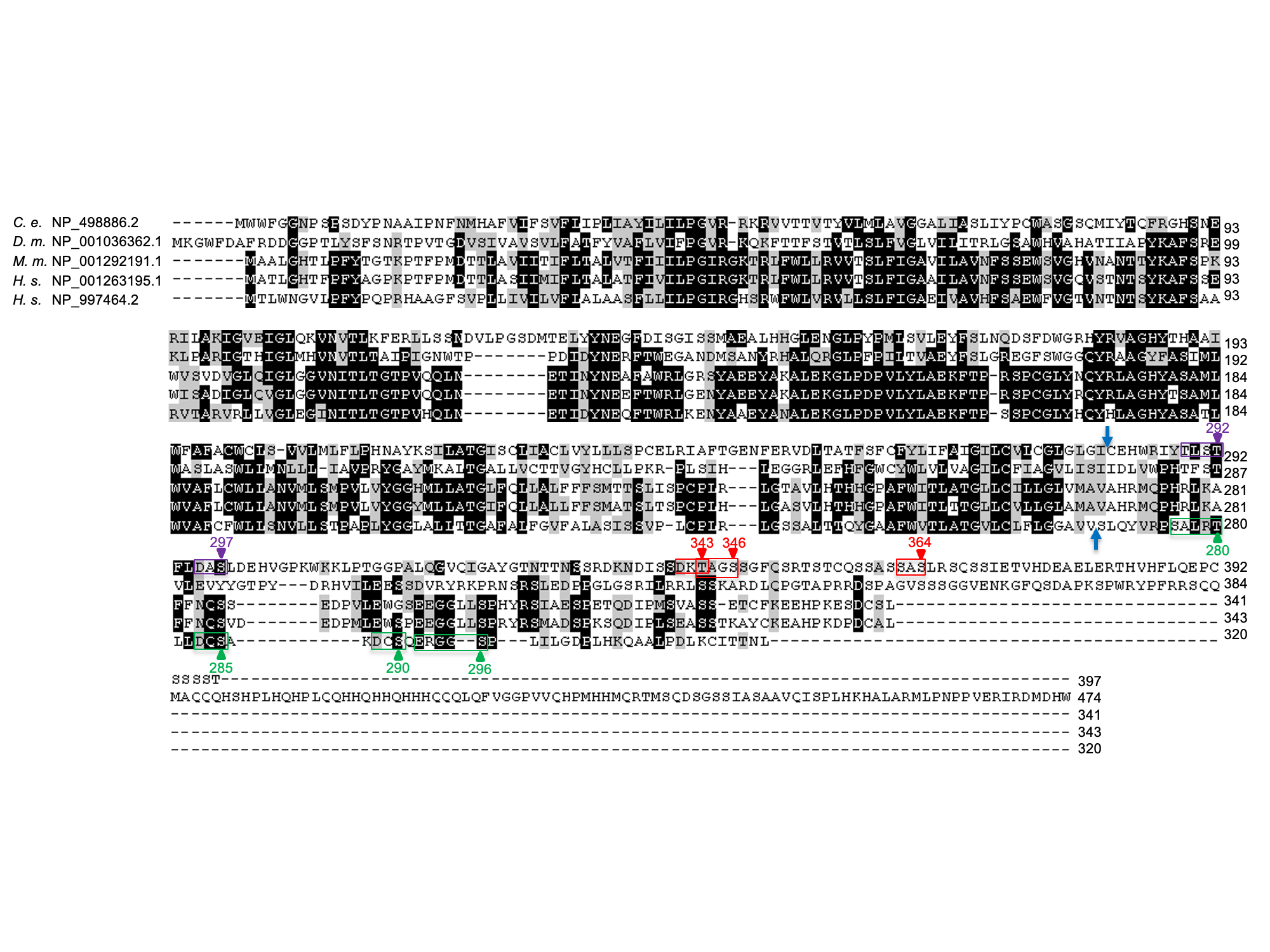

Supplement: S8 Fig — Top three predicted (scansite4.mit.edu) CSNK1 phosphorylation sites (similar to the conserved D/E/(p)S/T(X)1-3S/T sequence) in DOXA-1 C-terminal region (start indicated by blue arrow above, predicted by uniprot.org) are enclosed in red boxes with the phospho-acceptor pointed out by red arrowheads. Four potential CSNK1 phosphorylation sites in DUOXA2 C-terminal region (start indicated by blue arrow below, predicted by uniprot.org) are enclosed in green boxes with the phospho-acceptor pointed out by green arrowheads. Two of these sites appear to be conserved in DOXA-1 (purple box and purple arrowheads). C.e.: C. elegans DOXA-1; D.m.: Drosophila mol-PF; M.m.: mouse DUOXA1; H.s.: human DUOXA1 and DUOXA2. (TIFF) [file pgen.1010740.s008.tiff]

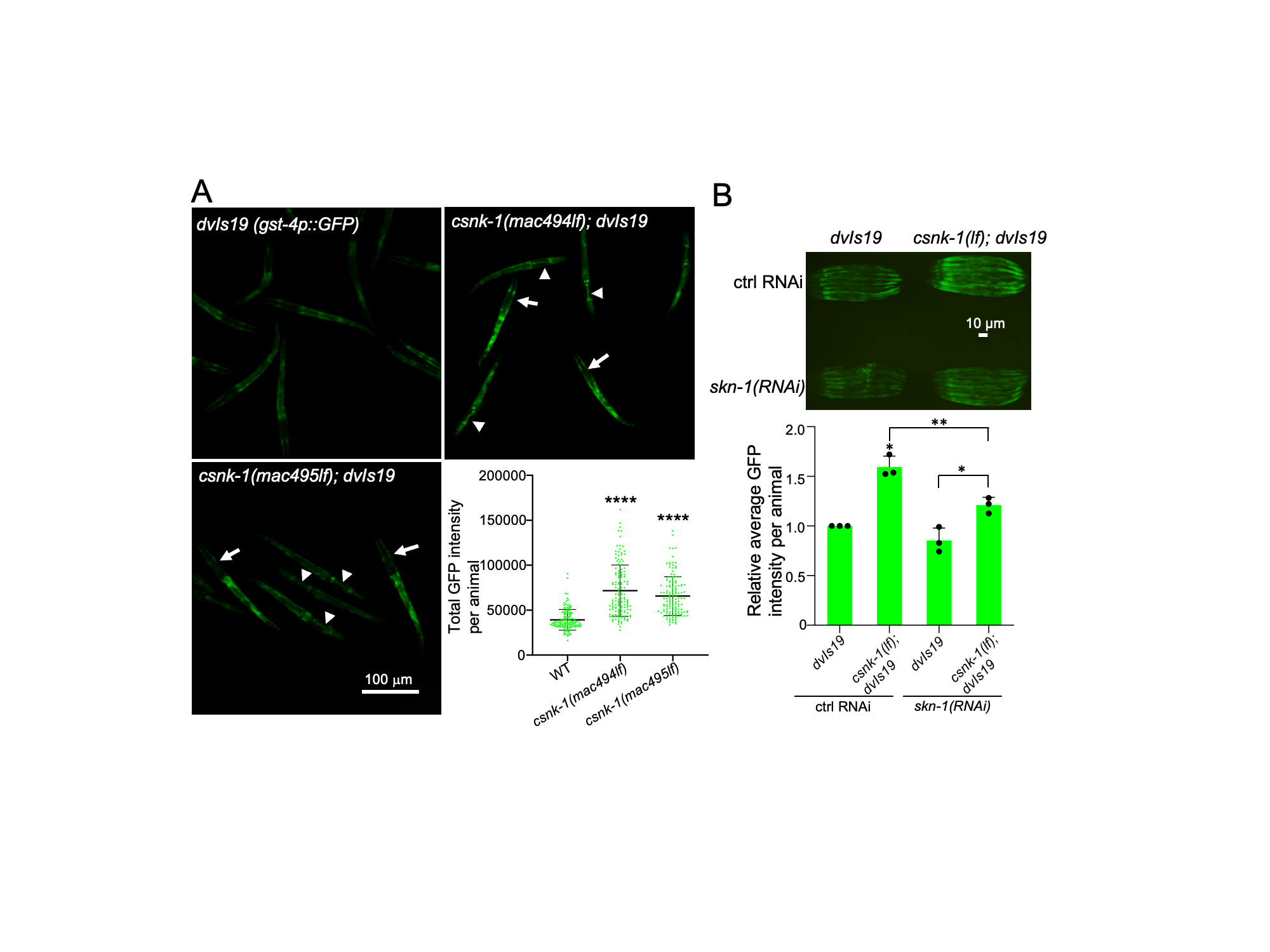

Supplement: S9 Fig — (A) Synchronized L1 animals of the indicated genotypes were observed after 4 hrs on food. For csnk-1(lf) animals, a mixed population of csnk-1(lf)/hT2; dvIs19 and csnk-1(lf); dvIs19 were shown, in which the csnk-1(lf)/hT2 genotype can be identified by pharyngeal GFP signals (arrowheads) expressed from an integrated GFP reporter in hT2 balancer. Animals without pharyngeal GFPs were identified as csnk-1(lf) homozygous (arrows). Pictures were taken with the same exposure time of 100 ms. Total GFP intensity of individual animal was measured using ImageJ and compared with those of dvIs19 controls. Results were based on over 130 individuals for each genotype. Statistics: two-tailed unpaired Student’s t-test. ***: p < 0.001. (B) Effects of csnk-1(lf) on dvIs19 expression with or without skn-1(RNAi). Animals were treated with feeding RNAi for 72 hrs after hatching. For each experiment, seven animals were aligned and measured for total GFP intensities using ImageJ. The average GFP intensity per animal was adjusted to that of dvIs19; ctrl RNAi group. Pictures were taken with the same exposure time of 100 ms. Results were based on three biological replicates. Statistics: two-tailed unpaired Student’s t-test. *: p < 0.05; **: p < 0.01. (TIFF) [file pgen.1010740.s009.tiff]

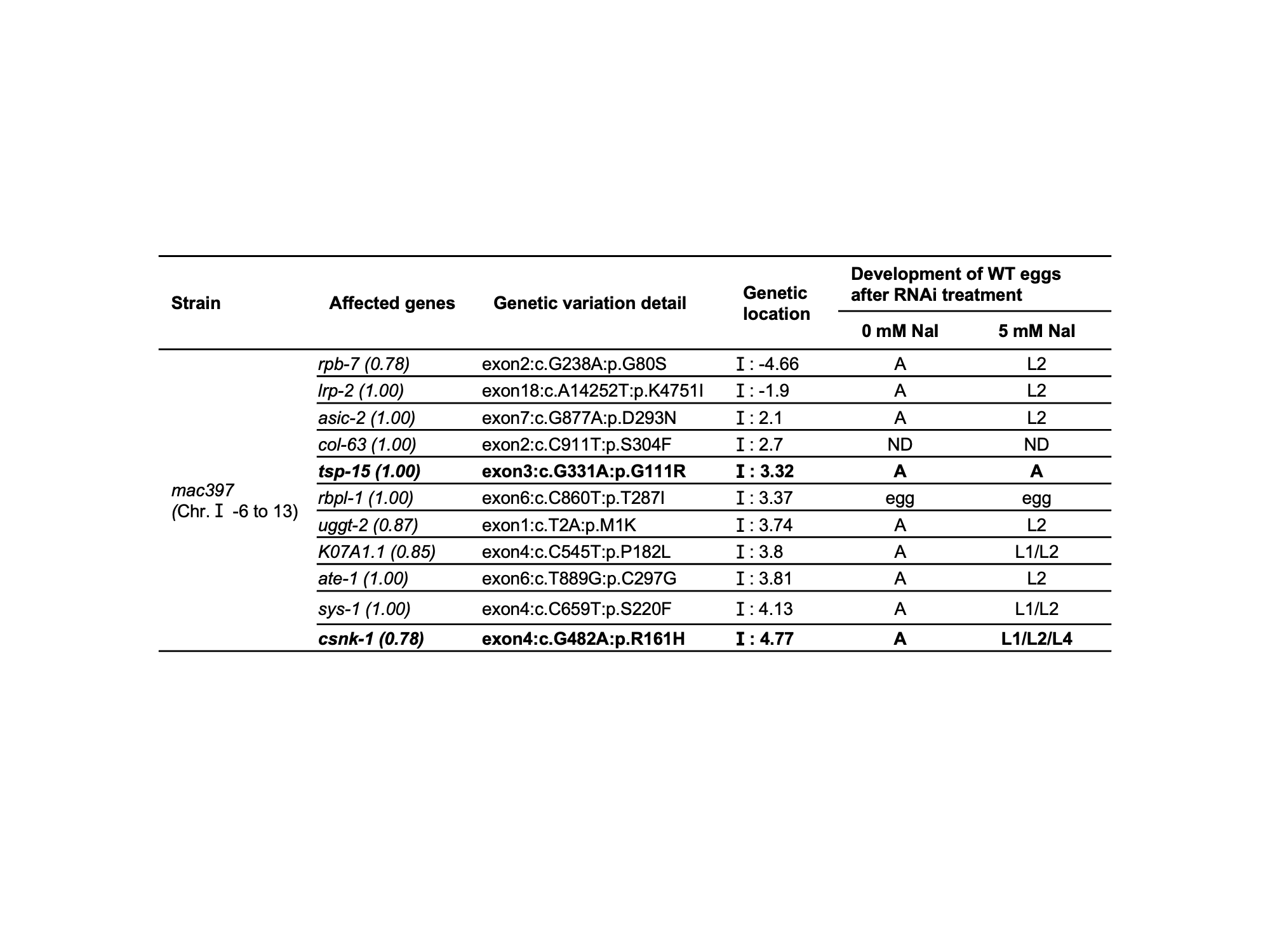

Supplement: S1 Table — Ratios of mutated sequences are shown in parentheses. A ratio of 1.00 suggests homozygous, and a ratio less than 1.00 suggests heterozygous. A: adults. ND: not determined. (TIFF) [file pgen.1010740.s010.tiff]

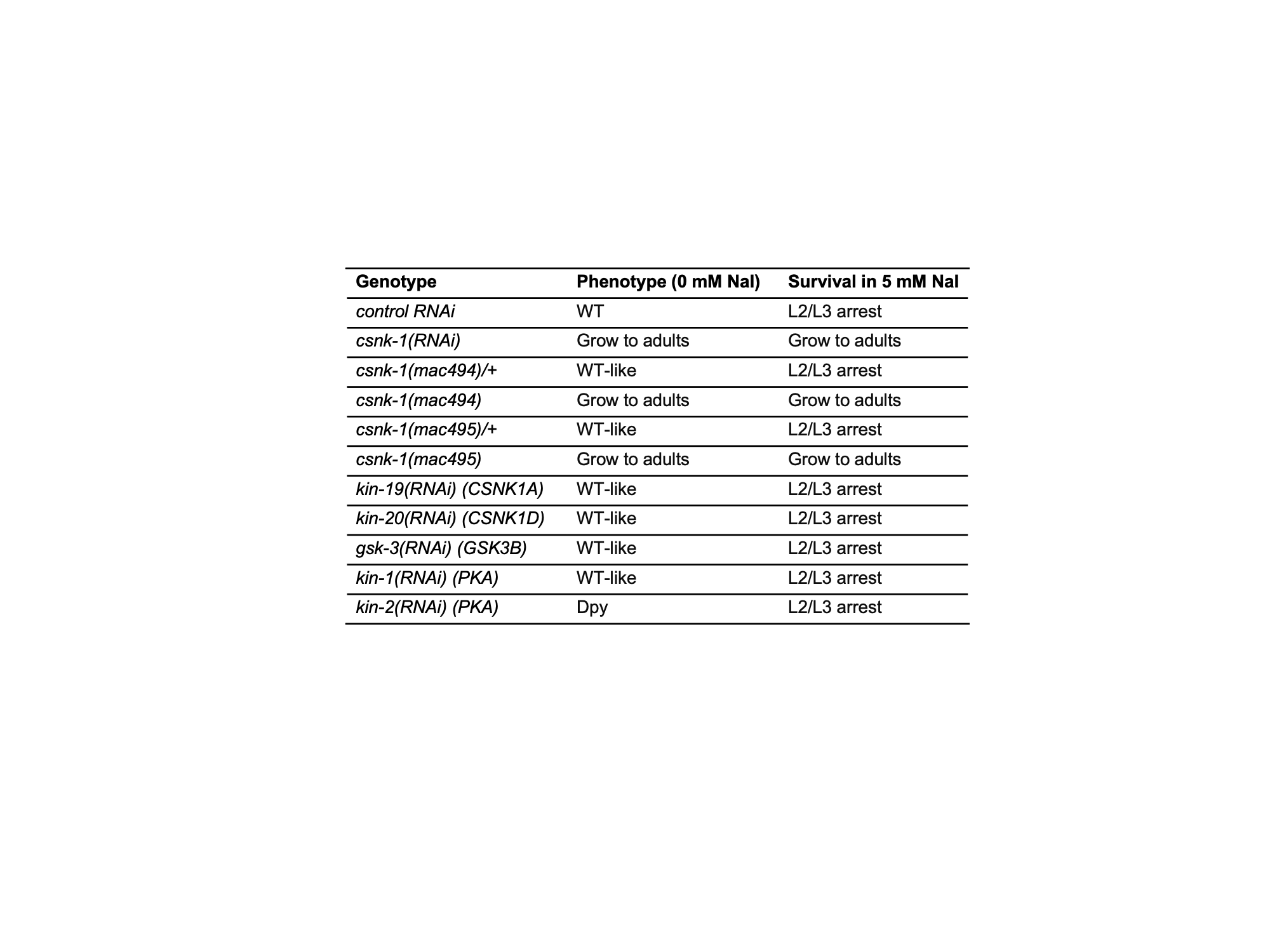

Supplement: S2 Table — Mammalian orthologs or homologs are indicated in the parentheses. (TIFF) [file pgen.1010740.s011.tiff]

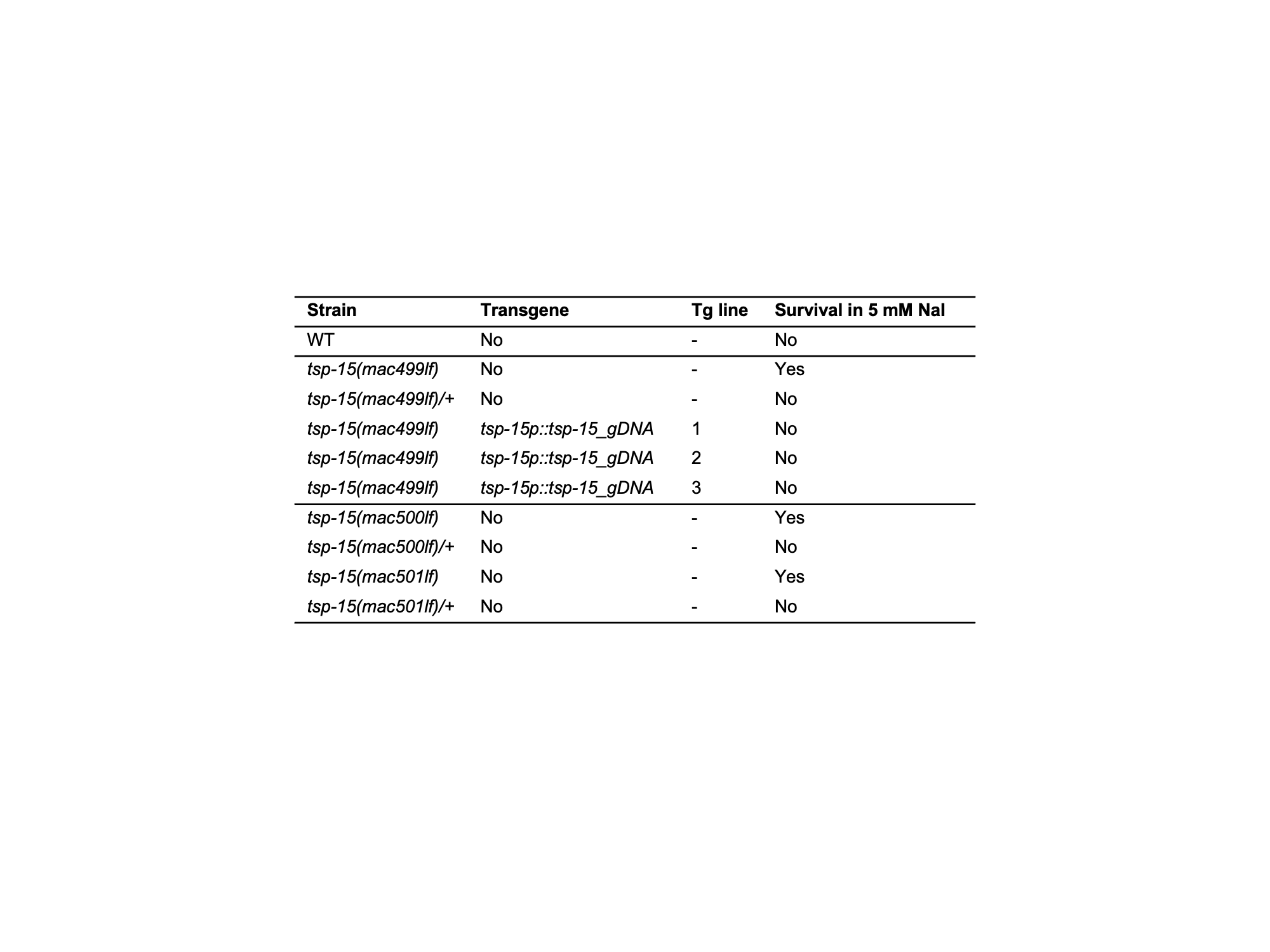

Supplement: S3 Table — tsp-15(mac500lf) and tsp-15(mac501lf) are knockin genocopies of tsp-15(mac499lf) generated using the CRISPR/Cas9 method. (TIFF) [file pgen.1010740.s012.tiff]

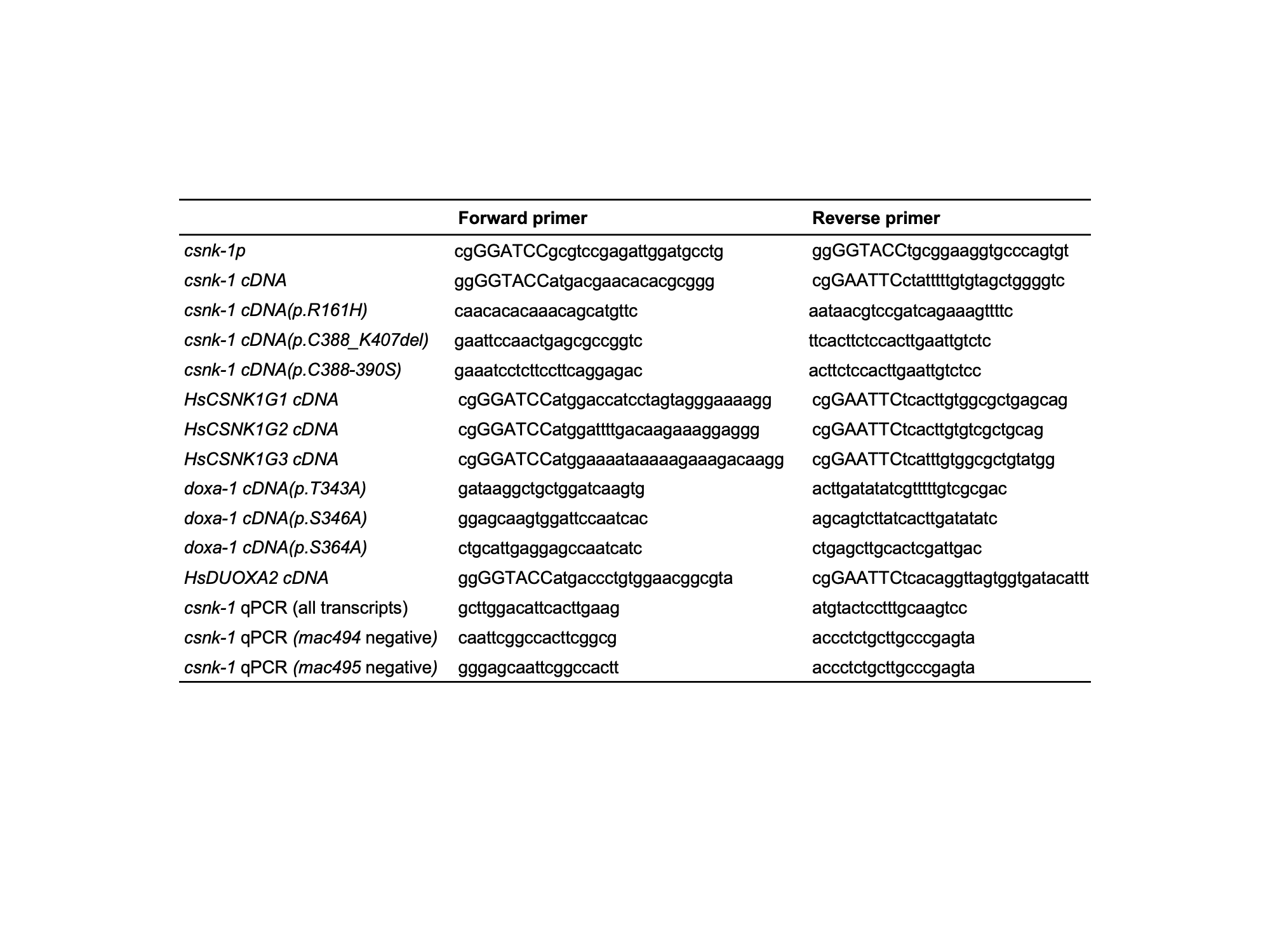

Supplement: S4 Table — Restriction sites are shown in uppercase. (TIFF) [file pgen.1010740.s013.tiff]

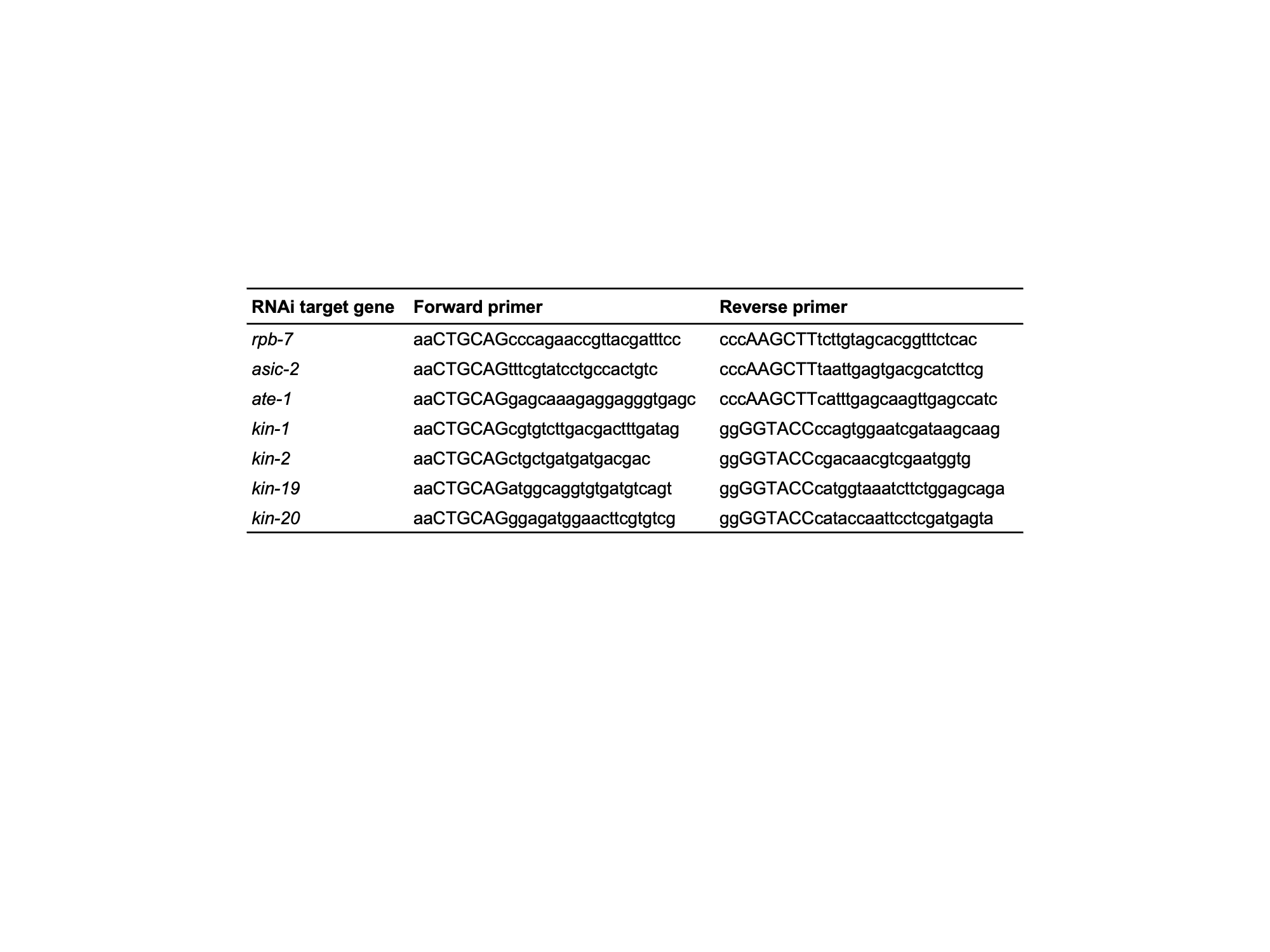

Supplement: S5 Table — Restriction sites are shown in uppercase. (TIFF) [file pgen.1010740.s014.tiff]

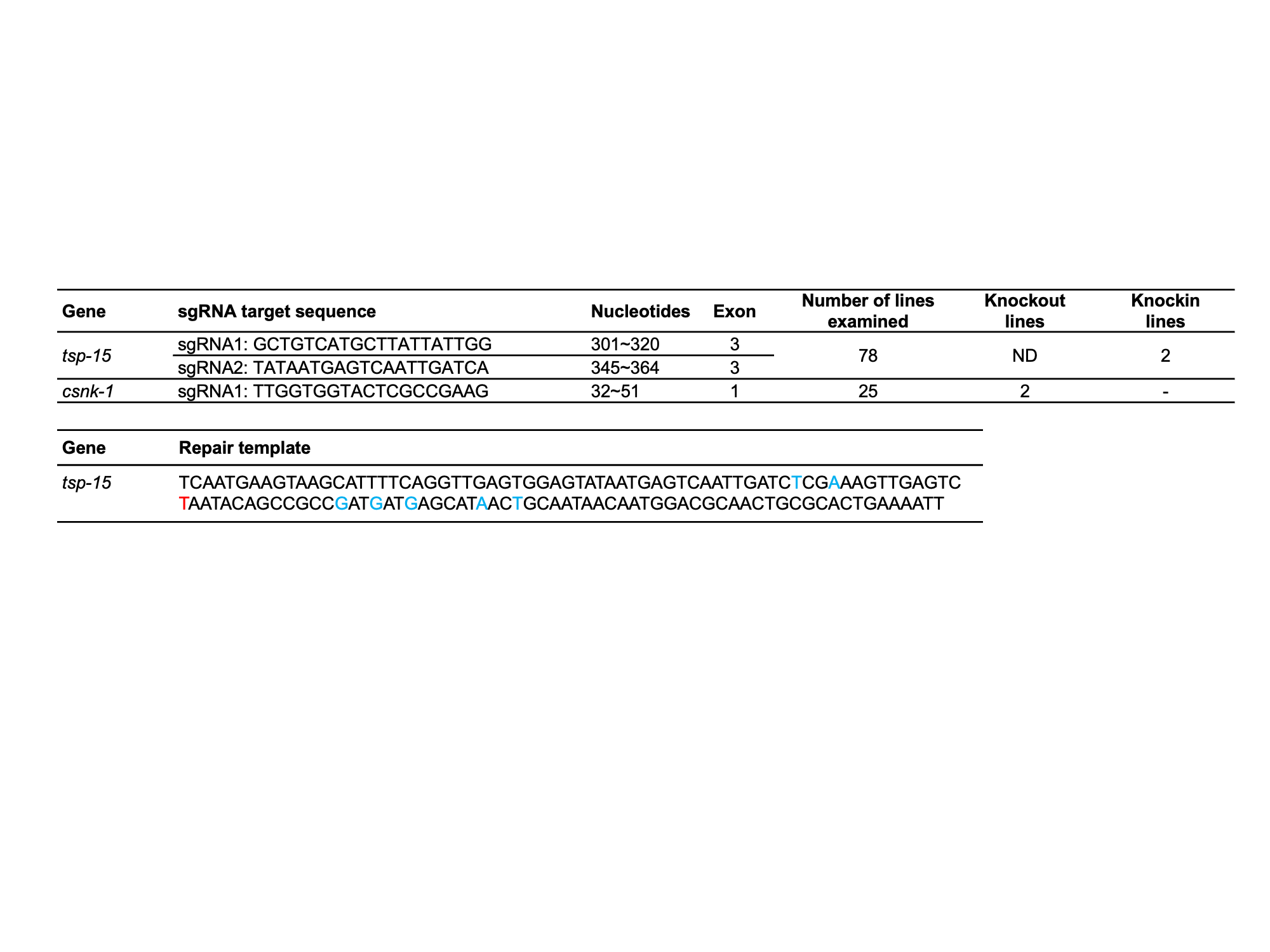

Supplement: S6 Table — ND: not determined. For tsp-15 repair template, the letter in red was for introducing the missense mutation and letters in blue were for introducing silent mutations. (TIFF) [file pgen.1010740.s015.tiff]
