# Supplementary material for: Casein kinase 1 gamma regulates oxidative stress response via interacting with the NADPH dual oxidase complex
Source: PLoS Genet. 2023 Apr 26;19(4):e1010740. doi: 10.1371/journal.pgen.1010740 (PMC10166522; doi:10.1371/journal.pgen.1010740)
Supplement: S1 Text — (DOCX) [file pgen.1010740.s016.docx]

**Strains**

*C. elegans* animals were grown on *E. coli* OP50 at 20 °C as described [1], unless otherwise indicated. N2 (Bristol) was the reference wild type strain. Strains and mutations used in this study include:

CB4856 (Hawaiian) [2]

CB767 *bli-3(e767lf) I* [1]

CL2166 *dvIs19[pAF15(gst-4p::GFP::NLS)]* [3]

XW18042 *qxIs722[dpy-7p::dpy-7::SfGFP (single-copy)]* [4]

CSM637 *skn-1(lax120gf) IV* [5] (outcrossed from SPC167: *dvIs19 III; skn-1(lax120) IV*) [6]

JV1 *unc-119(ed3) III; jrIs1[rpl-17p::HyPer + unc-119(+)]* [7]

CSM627 *bli-3(mac40lf) I* [8]

CSM681 *tsp-15(mac33lf) I* [8]

CSM684 *skn-1(mac53gf) IV* [5]

CSM752 *doxa-1(mac55lf) III* [5]

CSM753 *doxa-1(mac67lf) III* [5]

CSM670 *csnk-1(mac397)* [5]

CSM638 *skn-1(zu135lf) IV/nT1[qIs51] (IV; V)* [5] (outcrossed from EU31: *skn-1(zu135) IV/nT1[unc-?(n754) let-?](IV; V)*) [9]

CSM1124 *csnk-1(mac494lf)/hT2[qIs48] I; +/hT2[qIs48] III*

CSM1125 *csnk-1(mac495lf)/hT2[qIs48] I; +/hT2[qIs48] III*

CSM1199 *tsp-15(mac499lf) I*

CSM1200 *tsp-15(mac500KI) I*

CSM1201 *tsp-15(mac501KI) I*

CSM1212 *bli-3(mac40lf) csnk-1(mac494lf)/hT2[qIs48] I; +/hT2[qIs48] III*

CSM1213 *bli-3(mac40lf) csnk-1(mac495lf)/hT2[qIs48] I; +/hT2[qIs48] III*

CSM1214 *csnk-1(mac494lf)/hT2[qIs48] I; +/hT2[qIs48] III; dvIs19 III*

CSM1215 *csnk-1(mac495lf)/hT2[qIs48] I; +/hT2[qIs48] III; dvIs19 III*

CSM1218 *csnk-1(mac494lf)/hT2[qIs48] I; +/hT2[qIs48] III; skn-1(mac53gf) IV*

CSM1219 *csnk-1(mac495lf)/hT2[qIs48] I; +/hT2[qIs48] III; skn-1(mac53gf) IV*

CSM1220 *csnk-1(mac494lf)/hT2[qIs48] I; +/hT2[qIs48] III; skn-1(lax120gf) IV*

CSM1221 *csnk-1(mac495lf)/hT2[qIs48] I; +/hT2[qIs48] III; skn-1(lax120gf) IV*

CSM1257 *bli-3(e767lf) csnk-1(mac494lf)/hT2[qIs48] I; +/hT2[qIs48] III*

CSM1258 *bli-3(e767lf) csnk-1(mac495lf)/hT2[qIs48] I; +/hT2[qIs48] III*

CSM1259 *csnk-1(mac494lf)/hT2[qIs48] I; doxa-1(mac55lf)/hT2[qIs48] III*

CSM1260 *csnk-1(mac495lf)/hT2[qIs48] I; doxa-1(mac55lf)/hT2[qIs48] III*

CSM1451 *csnk-1(mac494lf)/hT2[qIs48] I; +/hT2[qIs48] III; qxIs722 II*

CSM1452 *csnk-1(mac494lf)/hT2[qIs48] I; +/hT2[qIs48] III; jrIs1*

**Plasmids**

To construct the *csnk-1p::GFP* plasmid, a *csnk-1* promoter (3.1 kb upstream of the *csnk-1* start codon) was amplified by PCR and subcloned to the pPD95_79 vector (a gift from Andrew Fire) using *BamH*I and *Kpn*I sites.

To construct the *csnk-1p::csnk-1*_*cDNA* plasmid, the *csnk-1* cDNA fragment was amplified from first strand cDNAs and used to replace *GFP* of *csnk-1p::GFP* using *Kpn*I and *EcoR*I sites.

To construct the *dpy-7p::csnk-1_cDNA* plasmid, a *dpy-7* promoter (430 bp upstream of the *dpy-7* start codon) [10] was subcloned to the pPD95_79 vector using *Hind*III and *BamH*I sites. The *csnk-1* cDNA fragment was subcloned to the pPD95_79-*dpy-7p* backbone using *Kpn*I and *EcoR*I sites.

To construct the *nhx-2p:: csnk-1_cDNA* plasmid, an *nhx-2* promoter (4.1 kb upstream of the *nhx-2* start codon) [11] was subcloned to pPD95_79 using *Pst*I and *Xma*I sites. The *csnk-1* cDNA fragment was subcloned to the pPD95_79-*nhx-2p* backbone using *Kpn*I sites.

To construct the *dpy-7p::HsCSNK1G1/2/3_cDNA* plasmids, the *HsCSNK1G* cDNA fragments were amplified from a human cDNA preparation and subcloned to the pPD95_79-*dpy-7p* backbone using *BamH*I and *EcoR*I sites.

To construct the *csnk-1p::csnk-1*_*cDNA(p.C388-390S)*, *csnk-1p::csnk-1_cDNA(p.C388_K407del)* and *csnk-1p::csnk-1*_*cDNA(mac397)* plasmids, the aforementioned *csnk-1p::csnk-1*_*cDNA* plasmid was mutated by Q5 Site-Directed Mutagenesis Kit (New England Biolabs).

To construct the *dpy-7p:: csnk-1_cDNA::mCherry* plasmid, the *mCherry* coding fragment was amplified from pCFJ90 (*myo-2p::mCherry*) [12] and subcloned to the aforementioned *dpy-7p::csnk-1_cDNA* plasmid using NEBuilder HiFi DNA Assembly kit (New England Biolabs).

To construct the *dpy-7p::doxa-1cDNA::GFP* plasmid, a *doxa-1* cDNA fragment was subcloned to the pPD95_79-*dpy-7p* backbone using *BamH*I and *Age*I sites.

To generate the *doxa-1p::doxa-1*_*cDNA(p.T343A),* *(p.S346A)* or *(p.S364A)* construct, the *p.T343A, p.S346A* or *p.S364A* mutation was introduced to a previously described *doxa-1* rescue construct [5] using Q5 Site-Directed Mutagenesis Kit (New England Biolabs).

To construct the *pcDNA3.1-doxa-1_cDNA::HA* plasmid, a *doxa-1* cDNA fragment was subcloned to the *pcDNA3.1* backbone using *Kpn*I and *EcoR*I sites. *HA* tag was introduced using Q5 Site-Directed Mutagenesis Kit (New England Biolabs).

To construct the *pCMV-Tag2B(FLAG)::csnk-1_cDNA* plasmid, a *csnk-1* cDNA fragment was subcloned to the *pCMV-Tag2B* backbone using *BamH*I and *EcoR*I sites.

To construct *pcDNA3.1-HsDUOXA2_cDNA::HA* plasmid, a human *DUOXA2* cDNA fragment was subcloned into the *pcDNA3.1* backbone using *Kpn*I and *EcoR*I sites. *HA* tag was introduced using Q5 Site-Directed Mutagenesis Kit (New England Biolabs).

To construct the *pCMV-Tag2B(FLAG)::HsCSNK1G2_cDNA* plasmid, a human *CSNK1G2* cDNA fragment was subcloned to the *pCMV-Tag2B* backbone using *BamH*I and *EcoR*I sites.

To construct the *pCold TF::doxa-1_cDNA* plasmid, a *doxa-1* full-length cDNA fragment was subcloned to the *pCold TF DNA* backbone using *Kpn*I and *BamH*I sites.

To construct the *pCold TF::csnk-1_cDNA* plasmid, a *csnk-1* full-length cDNA fragment was subcloned to the *pCold TF DNA* backbone using *BamH*I and *Hind*III sites.

PCR primers are listed in Table S4.

We generated RNAi constructs for *rpb-7, asic-2, ate-1*, *kin-1, kin-2, kin-19,* or *kin-20* by subcloning a 400-1000 bp genomic fragment of each gene into the pPD129_36 (L4440, a gift from Andrew Fire, Addgene plasmid # 1654) vector using *Pst*I and *Hind*III sites respectively. PCR primers are listed in Table S5.

Plasmids were verified by restriction digestion and sequencing.

**References:**

1. Brenner S. The genetics of *Caenorhabditis elegans*. Genetics. 1974;77: 71–94.

2. Wicks SR, Yeh RT, Gish WR, Waterston RH, Plasterk RH. Rapid gene mapping in *Caenorhabditis elegans* using a high density polymorphism map. Nat Genet. 2001;28: 160–164. doi:10.1038/88878

3. Link CD, Johnson CJ. Reporter transgenes for study of oxidant stress in *Caenorhabditis elegans*. Methods Enzymol. 2002;353: 497–505. doi:10.1016/s0076-6879(02)53072-x

4. Miao R, Li M, Zhang Q, Yang C, Wang X. An ECM-to-Nucleus Signaling Pathway Activates Lysosomes for *C. elegans* Larval Development. Dev Cell. 2020;52: 21-37.e5. doi:10.1016/j.devcel.2019.10.020

5. Xu Z, Hu Y, Deng Y, Chen Y, Hua H, Huang S, et al. WDR-23 and SKN-1/Nrf2 Coordinate with the BLI-3 Dual Oxidase in Response to Iodide-Triggered Oxidative Stress. G3 (Bethesda). 2018;8: 3515–3527. doi:10.1534/g3.118.200586

6. Paek J, Lo JY, Narasimhan SD, Nguyen TN, Glover-Cutter K, Robida-Stubbs S, et al. Mitochondrial SKN-1/Nrf mediates a conserved starvation response. Cell Metab. 2012;16: 526–537. doi:10.1016/j.cmet.2012.09.007

7. Back P, De Vos WH, Depuydt GG, Matthijssens F, Vanfleteren JR, Braeckman BP. Exploring real-time in vivo redox biology of developing and aging *Caenorhabditis elegans*. Free Radic Biol Med. 2012;52: 850–859. doi:10.1016/j.freeradbiomed.2011.11.037

8. Xu Z, Luo J, Li Y, Ma L. The BLI-3/TSP-15/DOXA-1 dual oxidase complex is required for iodide toxicity in *Caenorhabditis elegans*. G3 (Bethesda). 2014;5: 195–203. doi:10.1534/g3.114.015982

9. Bowerman B, Eaton BA, Priess JR. *skn-1*, a maternally expressed gene required to specify the fate of ventral blastomeres in the early *C. elegans* embryo. Cell. 1992;68: 1061–1075. doi:10.1016/0092-8674(92)90078-q

10. Gilleard JS, Barry JD, Johnstone IL. *cis* regulatory requirements for hypodermal cell-specific expression of the *Caenorhabditis elegans* cuticle collagen gene *dpy-7*. Mol Cell Biol. 1997;17: 2301–2311. doi:10.1128/MCB.17.4.2301

11. Nehrke K. A Reduction in Intestinal Cell pH i Due to Loss of the *Caenorhabditis elegans* Na+/H+ Exchanger NHX-2 Increases Life Span *. Journal of Biological Chemistry. 2003;278: 44657–44666. doi:10.1074/jbc.M307351200

12. Frøkjaer-Jensen C, Davis MW, Hopkins CE, Newman BJ, Thummel JM, Olesen S-P, et al. Single-copy insertion of transgenes in *Caenorhabditis elegans*. Nat Genet. 2008;40: 1375–1383. doi:10.1038/ng.248
