# Supplementary material for: Casein kinase 1 gamma regulates oxidative stress response via interacting with the NADPH dual oxidase complex
Source: PLoS Genet. 2023 Apr 26;19(4):e1010740. doi: 10.1371/journal.pgen.1010740 (PMC10166522; doi:10.1371/journal.pgen.1010740)
Supplement: Attachment — Submitted filename: CSNK-1 review.docx [file pgen.1010740.s018.docx]

In the article entitled “Casein kinase 1 gamma regulates oxidative stress response via interacting with the NADPH dual oxidase complex”, the authors reported an involvement of ‘dark kinase’ CSNK1Gs in oxidative stress response for the first time. By combination of genetic and cell biological approaches, the authors suggested that C. elegans CSNK-1 is involved in ROS production by interaction with dual oxidase complex (BLI-3/TSP-15/DOXA-1). The authors identified mutation in *csnk-1* gene resulted in resistance to excessive NaI stress. The *csnk-1* mutants were interacted genetically with genes composing dual oxidase complex, which are previously reported by the authors. The authors also suggested that CSNK-1 participates in regulation of dual oxidase complex by phosphorylation of dual oxidase maturation factor (DOXA-1), and this interaction is conserved in the mammalian orthologues, between DUOXA2 and CSNK1G2.

Overall, their genetic experiments were carefully and honestly performed. In particular, identification of *mac397* allele was impressive, since *tsp-15* mutation which was already shown to cause NaI-resistance, was also found in the same mutant. On the other hand, cell biological analysis and discussion seems to be insufficient to support their conclusion. Therefore, this article is not allowed to accept in current form due to several critical points to revise as indicated below.

Major points:

1. The authors produced several of *csnk-1(lf)* mutants, but it is not clear that these are null or hypomorph. This clarification will explain different phenotype severity shown in Fig.S2. The protein or RNA expression levels in these mutants could be investigated by Western blot or qRT-PCR. As shown in Fig.S1, the DNA sequences of *csnk-1* and *CSNK1Gs* are highly conserved, so anti-CSNK1G antibody may cross to CSNK-1 protein. Anti-CSNK-1 antibody reported in Panbianco et al (PMID18694560) may be also available.
2. Fig.2E: I have a concern about this pull-down experiment, since it is questionable that membrane protein DOXA-1 has correct topology in bacterial protein expression system. Therefore, I’m not very convinced that the interaction with CSNK-1 is physiologically occurred.
3. Fig.3: The authors did not mention why did they focus on CSNK1G2 among CSNK1Gs in interaction assay with DUOXA2. Is this because the results of transgene assay shown in Table 1?
4. Fig.3C and 3D: Pan-CK1 inhibitor D4476 decreased ROS production in HEK293 cell. However, this data does not support interaction of CSNK1G with dual oxidase complex since endogenous expression of these genes are unknown. Exogenous over-expression of CSNK1G2 and DUOXA2 resulted in increase of ROS generation, and the promotion was suppressed by D4476. However, it remains still unclear that which molecule is responsible for the ROS production in this assay. Therefore, these data seem to be insufficient to support the author’s argument.
5. Fig.S6: Threonine at position 343 of DOXA-1 is not conserved in human DUOXA2. If DUOXA2 is regulated via phosphorylation by CNSK1G, where is possible phosphorylation site of DUOXA2? The authors indicated direct interaction of DUOXA2 with CNSK1G2, but if they argue conservation of regulatory mechanism of CNSK1G in DUOX-mediated ROS production, they should discuss about this point.
6. Fig.4 and Table 4, 5: The results of genetic interaction of *cnsk-1* with *skn-1* were complicated. I feel that these results should be presented in supplemental data for now. It is reasonable that *csnk-1(lf) skn-1(gf)* double mutants showed high tolerance to NaI stress. However, the authors did not explain clearly the reason why *csnk-1(lf)* mutation increased NaI survival rate in *skn-1(lf)* heterozygous background. More additional studies should be provided to solve the problems. For example, investigation of ROS levels in the double mutants may give helpful data to explain this phenotype.
7. Throughout this manuscript, the author suggested that phosphorylation of DOXA-1 by CSNK-1. However, direct evidence of phosphorylation of DOXA-1 was not presented. It is also still unknown whether the phosphorylation is dependent on CSNK-1. For example, these issues may be addressed by Western blot analysis of DOXA-1 protein in *cnsk-1(lf)* mutants by the use of Phos-tag (example of use PMID: 29330450).

Minor points:

1. The authors described the general functions of CSNK1G in Discussion, but they should briefly mention them in Introduction for unfamiliar readers.
2. The authors produced *mac494* and *mac495*, loss-of-function (lf) alleles of *csnk-1*, which may result in frameshift mutation in *csnk-1* gene. Why didn’t the authors produce CSNK-1 R161H knock-in, like they did in *tsp-15* mutant (*mac500* and *mac501*)?
3. Fig.2 and Fig.3: The authors should indicate molecular weight in Western blot analysis.
4. Fig.3: The authors should use higher magnification images of immunofluorescent study to show colocalization of DUOXA2 and CSNK1G2 clearly.
5. Fig.S3 and S4: These figures indicate the same results. The authors should use Normaski DIC images of Fig.S4 to show the phenotypes of double mutants, since detail morphology are illegible in Fig.S3 resolution. Also, individual animal should be shown by DIC images with higher magnification. In addition, representative images of double mutants of *tsp-15* and *csnk-1* should be presented.
6. Table S3 and Table 5: The authors should describe the results of NaI-resistance assay by numbers or ratio like Table 1, not by simple Yes/No definition.
