# Supplementary material for: Casein kinase 1 gamma regulates oxidative stress response via interacting with the NADPH dual oxidase complex
Source: PLoS Genet. 2023 Apr 26;19(4):e1010740. doi: 10.1371/journal.pgen.1010740 (PMC10166522; doi:10.1371/journal.pgen.1010740)
Supplement: Attachment — Submitted filename: ResponseToReviewers.docx [file pgen.1010740.s019.docx]

**Response to Reviewer #1**

1. However, the details of the approach, including the number of animals, replicates, and the statistical measure used are not adequately described.

**Response:** We provided the details in methods, figure legends, table notes and raw data in the revised manuscript.

2. It is important for the authors to find a more appropriate method to measure ROS, such as the Amplex red, in vivo HyPer (Fig. 2G and H) reporter which is specific to hydrogen peroxide.

**Response:** We measured ROS levels using Amplex Red assay and crossed a Hyper reporter (*jrIs1[rpl-17p::HyPer + unc-119(+)]*) generated by Back et al (2012) to *csnk-1(lf)* mutants. These results are now presented in Fig. 2I, 2J and 2K and they are consistent with the DCFDA results.

We also used Amplex Red to measure the effects of D4476 on ROS levels in HEK293T cells and observed similar effect like that using DCFDA. The result is presented in Fig. 3D.

3. As presented, there is confusion whether the intestine or the hypodermis/epidermis are key in the observed response. This is due to the fact that the data in Table 1 does not support the claims made in the text. It shows that the intestinal expression of *csnk-1* rescues survival and not epidermis expression.

**Response:** Wildtype animals failed to survive in 5 mM NaI (0% survival rate), while *csnk-1(lf)* mutants have ~79% survival rate. Therefore, the ability to survive in NaI is the mutant phenotype. When *csnk-1* wt transgenes were expressed in the epidermis of *csnk-1(lf)* mutants, the survival rate of these animals was reduced to 0.5-3%, which is close to that of wildtype animals, suggesting a rescue effect. However, *csnk-1* WT transgenes expressed in the intestine of *csnk-1(lf)* mutants had no such effect, suggesting no rescue. Together these results suggest a function of epidermal CSNK-1 in causing the lack of survival of wildtype animals in NaI. To clarify the description of the phenotype, we abbreviated the mutant “survived-in-sodium-iodide” phenotype to “Sisi” in the revised manuscript, hoping this change causes less confusion for readers.

4. In general, experiments need to have appropriate statistical comparisons applied and detailed in the figure legends.

**Response:** We performed relevant statistical analyses on all data and explained the details of the methods, number of replicates and number of animals in figure legends, where applicable.

5. Table 1/3/4/5 needs to include replicates and p-values

**Response:** We provided these details in the revised manuscript.

6. Data in Figure S2 needs more data than one replicate with a N=3.

**Response:** We performed more experiments and now each bar graph is based on at least 3 replicates. The results are now presented as Figure S3. The details are provided in figure legends.

7. The data as presented in Figure 4b is not appropriately quantified.

**Response:** We quantified GFP intensities of three replicate groups of animals for each treatment and performed statistical analyses. Fig. 4 is now presented as Fig. S9 considering Reviewer 2’s suggestion and the preliminary nature of the analysis.

8. Methodologically the data in Figure 4 seems to be censored by brightness. This quantification needs to be explicitly detailed as it would appear that only the brightest worms are being chosen for analysis as the representative worms. If this is incorrect, the authors should clarify in the methods section how these measurements were performed. In addition, Figure 4a the authors should state what strain or condition the GFP intensity is relative to?

**Response:** *csnk-1(lf)* mutations are maintained using *hT2[qIs48]* balancer, which expresses GFP in the pharynx. The pictures in the original Figure 4A showed synchronized populations containing both *csnk-1(lf)/hT2[qIs48]; dvIs19* and *csnk-1(lf); dvIs19* animals, among which *csnk-1(lf)* mutants were identified by the lack of pharyngeal GFP and *csnk-1(lf)/hT2[qIs48]* animals were identified by the presence of pharyngeal GFP. In these pictures, *csnk-1(lf); dvIs19* mutants exhibited brighter GFP signals, while *csnk-1(lf)/hT2; dvIs19* mutants were less bright. The pictures were taken using the same magnification and exposure. We measured total GFP intensity of each *csnk-1(lf); dvIs19* homozygous animals using ImageJ and compared with those of *dvIs19* controls. In the revised manuscript better pictures are presented to show the animals more clearly and representative animals are labeled. The information is clarified in the figure legend.

9. The authors demonstrate conservation of the interaction between DUOXA2 and CSNK1G2 in mammals, however, this data would be strengthened by expanding the imaging approach in Panel 3A to include more than 1 cell and by performing the reverse IP (IP FLAG and detect HA) to show the biochemical interaction.

**Response:** We performed new colocalization experiments between DUOXA2 and CSNK1G2 in HEK293T cells. Pictures containing several cells are shown in Fig. 3A. We also performed reverse IP and reverse pulldown between *C. elegans* CSNK-1 and DOXA-1 in HEK293T cells. The results are now presented in Fig. 2E and 2G. The specific interaction between *C. elegans* CSNK-1 and DOXA-1 is strengthened by the new reverse biochemical experiments.

We have not been able to perform reverse IP between DUOXA2 and CSNK1G2 due to pandemic interruption of our planned experiments. For this reason, our conclusion that the biochemical interaction between CSNK-1 and DOXA-1 is “conserved” in human cells is weakened to “potentially conserved”. As a piece of supporting evidence for colocalization between DUOXA2 and CSNK1G2, the original colocalization picture in the HeLa cell is now presented in Fig. S3I.

Minor Concerns:

1. Generally, images should include the relevant exposure times in the figure legend if different across experiments or in the methods section if universally applied.

**Response:** These details are provided in figure legends, where applicable.

**Response to Reviewer #2:**

1. The authors produced several of *csnk-1(lf)* mutants, but it is not clear that these are null or hypomorph. This clarification will explain different phenotype severity shown in Fig.S2. The protein or RNA expression levels in these mutants could be investigated by Western blot or qRT-PCR.

**Response:** *csnk-1(mac494)* and *csnk-1(mac495)* are frameshift mutations close to the start codon, which are predicted to cause multiple downstream stop codons in the mutant transcripts and to be potentially null. The more severe phenotype of *mac495* mutants or the less severe phenotype of *mac494* mutants might be related to a linked modifier mutation. Clarification of these possibilities would help us understand the phenotypic differences between the two mutants, which we propose to be addressed in future. Interestingly, the less severe molting defect of *mac494* mutant is an advantage as significantly more animals can be generated for the survival experiments.

We performed qRT-PCR experiments on synchronized L4 animals (Fig. S2). A primer pair amplifying all *csnk-1* transcripts found that both *csnk-1(lf)* mutants exhibited a reduction of total *csnk-1* transcripts and the reduction in *csnk-1(mac495)* mutants was more than that in *csnk-1(mac494)* mutants, which correlates with the phenotypic differences between the two mutants. Currently we do not have evidence supporting a causal relationship on this aspect. In addition, primer pairs specifically amplifying the wildtype *csnk-1* transcripts but not the mutant transcripts found that L4 *csnk-1(lf)* mutants express little or no wildtype transcripts. Therefore, maternal *csnk-1* transcripts are likely exhausted by the L4 larval stage.

2. As shown in Fig.S1, the DNA sequences of *csnk-1* and *CSNK1Gs* are highly conserved, so anti-CSNK1G antibody may cross to CSNK-1 protein. Anti-CSNK-1 antibody reported in Panbianco et al (PMID18694560) may be also available.

**Response:** We tested two CSNK1G2 antibodies, YT0649 (AA range 10-59 as immunogen, Immunoway) and sc-390970 (AA range 1-40 as immunogen, Santa Cruz). Both antibodies failed to detect CSNK-1 overexpressed in HEK293T cells. Antibodies binding the highly conserved kinase domain might not distinguish CSNK-1 vs other casein kinase 1 members.

Dr Ahringer also very kindly sent us the CSNK-1 antibody as reported in her publication (Panbianco et al, 2008). Unfortunately, China’s Customs denied clearance of the package and the antibody was eventually disposed of.

3. Fig.2E: I have a concern about this pull-down experiment, since it is questionable that membrane protein DOXA-1 has correct topology in bacterial protein expression system. Therefore, I’m not very convinced that the interaction with CSNK-1 is physiologically occurred.

**Response:** To address this concern, we performed reverse pulldown. We expressed HIS::TF::CSNK-1 in *E. coli* and purified the fusion protein. The purified HIS::TF::CSNK-1 can pull down DOXA-1::HA expressed in HEK293T cells. The result is shown in Fig. 2G. We also mentioned the possibility that protein misfolding might cause non-specific interactions (lines 282-284).

In addition, we performed reverse IP between CSNK-1 and DOXA-1 and detected similarly specific interaction. The result is shown in Fig. 2E.

4. Fig.3: The authors did not mention why did they focus on CSNK1G2 among CSNK1Gs in interaction assay with DUOXA2. Is this because the results of transgene assay shown in Table 1?

**Response:** It is correct that CSNK1G2 was chosen because its transgene appeared to have best rescuing effects. We pointed this out in the Results (lines 292-293).

5. Fig.3C and 3D: Pan-CK1 inhibitor D4476 decreased ROS production in HEK293T cell. However, this data does not support interaction of CSNK1G with dual oxidase complex since endogenous expression of these genes are unknown. Exogenous over-expression of CSNK1G2 and DUOXA2 resulted in increase of ROS generation, and the promotion was suppressed by D4476. However, it remains still unclear that which molecule is responsible for the ROS production in this assay. Therefore, these data seem to be insufficient to support the author’s argument.

**Response:** We agree with the analysis. D4476 is a pan-CSNK1 inhibitor. Its effect on ROS levels can be mediated by a CSNK1(s) other than CSNK1G. It may also have other targets. How much endogenous CSNK1G or DUOXA2 contributes to ROS levels in HEK293T cells is unknown before we understand the expression levels of different CSNK1s and DUOXAs and examine the effect of each one on ROS levels by knocking down or knocking out its expression. The D4476 results suggest that CSNK1s may affect ROS levels, but they are not sufficient to narrow down the effects to CSNK1Gs or DUOXA2. We clarified this point in the discussion (lines 390-395).

6. Fig.S6: Threonine at position 343 of DOXA-1 is not conserved in human DUOXA2. If DUOXA2 is regulated via phosphorylation by CNSK1G, where is possible phosphorylation site of DUOXA2? The authors indicated direct interaction of DUOXA2 with CNSK1G2, but if they argue conservation of regulatory mechanism of CNSK1G in DUOX-mediated ROS production, they should discuss about this point.

**Response:** Indeed DOXA-1 T343 is not conserved in DUOXA2. We visually scanned DUOXA2 C-terminal region and found four potential CSNK1 sites (now labeled in Fig. S8), two of which are probably conserved in DOXA-1 (also labeled in Fig. S8). We suggest future efforts should be taken to analyze these sites. We discussed these points (lines 397-407).

7. Fig.4 and Table 4, 5: The results of genetic interaction of *csnk-1* with *skn-1* were complicated. I feel that these results should be presented in supplemental data for now.

**Response:** We presented the original Fig. 4 as Fig. S9 in the revised manuscript considering that the gene expression analysis is limited. New figure panels and quantification of GFP intensities in RNAi-treated animals are also shown in the revised Fig. S9.

We hope to keep Tables 4 and 5 in the main text, feeling that the genetic interaction between *csnk-1* and *skn-1* is a natural extension of our earlier studies (Xu et al, 2018 and this study) and might be interesting to readers.

8. It is reasonable that *csnk-1(lf) skn-1(gf)* double mutants showed high tolerance to NaI stress. However, the authors did not explain clearly the reason why *csnk-1(lf)* mutation increased NaI survival rate in *skn-1(lf)* heterozygous background. More additional studies should be provided to solve the problems. For example, investigation of ROS levels in the double mutants may give helpful data to explain this phenotype.

**Response:** The genetic interaction between *csnk-1(lf)* and *skn-1(lf)* suggests that *csnk-1* is epistatic to *skn-1* for the survival phenotype. However, such an effect might be caused by *csnk-1* functioning downstream of or in parallel with *skn-1*, considering that survival in NaI can be caused by reducing ROS levels (like in *bli-3/tsp-15/doxa-1* mutants), increasing antioxidant expression (like in *skn-1(gf)* mutants) or using antioxidants like vitamin C or NAC (Xu et al, 2018)). Currently the molecular mechanism underlying their interactions remain unclear. We discussed these points (lines 412-421).

*skn-1(lf)* and *csnk-1(lf)* mutations are maintained using *nT1* and *hT2* balancers, respectively. We failed to generate a strain that produce double homozygous mutants for ROS analysis, likely because the two balancers cannot coexist in an animal. Double homozygous mutants derived from double heterozygotes need to be individually genotyped before being analyzed for ROS levels, which is technically challenging at this stage. We hope this problem will be addressed in the future.

9. Throughout this manuscript, the author suggested that phosphorylation of DOXA-1 by CSNK-1. However, direct evidence of phosphorylation of DOXA-1 was not presented. It is also still unknown whether the phosphorylation is dependent on CSNK-1. For example, these issues may be addressed by Western blot analysis of DOXA-1 protein in *cnsk-1(lf)* mutants by the use of Phos-tag (example of use PMID: 29330450).

**Response:** We agree with the analysis. We feel that to establish a direct kinase-substrate relationship between CSNK-1 and DOXA-1, which could also include experiments for comprehensively mapping and investigating the functions of phosphorylation sites using Phos-tag or other approaches, is a new project beyond the scope of the current study. To address the Reviewer’s concern, we specifically pointed out the lack of direct evidence that DOXA-1 is phosphorylated or is a substrate of CSNK-1 (lines 397-407).

**Minor points:**

1. The authors described the general functions of CSNK1G in Discussion, but they should briefly mention them in Introduction for unfamiliar readers.

**Response**: We introduced CSNK1G in the Introduction.

2. The authors produced *mac494* and *mac495*, loss-of-function (lf) alleles of *csnk-1*, which may result in frameshift mutation in *csnk-1* gene. Why didn’t the authors produce CSNK-1 R161H knock-in, like they did in *tsp-15* mutant (*mac500* and *mac501*)?

**Response:** We tried extensively to generate a genocopy of *mac397 (R161H)* mutation using the CRISPR/Cas9 but failed to obtain one.

3. Fig.2 and Fig.3: The authors should indicate molecular weight in Western blot analysis.

**Response**: Molecular weights are indicated.

4. Fig.3: The authors should use higher magnification images of immunofluorescent study to show colocalization of DUOXA2 and CSNK1G2 clearly.

**Response:** We performed new colocalization experiments in HEK293T cells and took higher resolution pictures. Clearer pictures with more cells are shown in Fig. 3A. The original colocalization picture is now shown as Fig. S3I.

5. Fig.S3 and S4: These figures indicate the same results. The authors should use Normaski DIC images of Fig.S4 to show the phenotypes of double mutants, since detail morphology are illegible in Fig.S3 resolution. Also, individual animal should be shown by DIC images with higher magnification. In addition, representative images of double mutants of tsp-15 and csnk-1 should be presented.

**Response:** Unfortunately, we are not equipped with Nomarski microscopy due to limited resources in our institute. In the revised Fig. S4, S5 and S6 (corresponding to original Fig. S3), we presented higher resolution pictures of these animals, in which the detailed morphologies can be seen. The original Fig. S4 is removed.

*tsp-15* (Chr I: 3.32) and *csnk-1* (Chr I: 4.77) are closely linked. We have tried extensively to recombine loss-of-function alleles of these genes to the same chromosome but failed to obtain one. Currently, we have yet to be able to show the morphology of *tsp-15(lf) csnk-1(lf)* double homozygous mutants.

6. Table S3 and Table 5: The authors should describe the results of NaI-resistance assay by numbers or ratio like Table 1, not by simple Yes/No definition.

**Response:** We quantified the survival rate in Table 5 and presented the new results. Table S3 was to test whether the isolated *tsp-15* mutation was dominant or recessive. We argue that Yes/No phenotype is sufficient for defining the mutation.
